# Effect of *Poria cocos* Mushroom Polysaccharides (PCPs) on the Quality and DNA Methylation of Cryopreserved Shanghai White Pig Spermatozoa

**DOI:** 10.3390/cells12111456

**Published:** 2023-05-24

**Authors:** Jinyong Zhou, Keqin Zhang, Jun Gao, Jiehuan Xu, Caifeng Wu, Mengqian He, Shushan Zhang, Defu Zhang, Jianjun Dai, Lingwei Sun

**Affiliations:** 1 Institute of Animal Husbandry & Veterinary Medicine, Shanghai Academy of Agricultural Sciences, Shanghai 201106, China; jinyzhou@126.com (J.Z.); 18141634280zk@gmail.com (K.Z.); gaojun@saas.sh.cn (J.G.); jiehuanxu810@163.com (J.X.); wucaifengwcf@163.com (C.W.); he1037247863@163.com (M.H.); smalltreexj@126.com (S.Z.); zhangdefuzdf@163.com (D.Z.); 2 College of Animal Sciences, Guizhou University, Guiyang 550025, China; 3 Key Laboratory of Livestock and Poultry Resources (Pig) Evaluation and Utilization, Ministry of Agriculture and Rural Affairs, Shanghai 201106, China

**Keywords:** Shanghai white pig, semen cryopreservation, *Poria cocos* mushroom polysaccharides (PCPs), antioxidant, DNA methylation

## Abstract

In this study, we explore the effects of *Poria cocos* mushroom polysaccharides (PCPs) on the quality and DNA methylation of the cryopreserved spermatozoa of Shanghai white pigs. A total of 24 ejaculates (three ejaculate samples per boar) from eight Shanghai white pigs were manually collected. The pooled semen was diluted with a based extender supplemented with different concentrations of PCPs (0, 300, 600, 900, 1200, and 1500 μg/mL). Once thawed, the quality of the spermatozoa and their antioxidant function were assessed. In the meantime, the effect of spermatozoa DNA methylation was also analyzed. The results show that compared with the control group, 600 μg/mL of PCPs significantly improves the spermatozoa viability (*p* < 0.05). The motility and plasma membrane integrity of the frozen–thawed spermatozoa are significantly higher after treatment with 600, 900, and 1200 μg/mL of PCPs compared with the control group (*p* < 0.05). In comparison with the control group, the percentages of acrosome integrity and mitochondrial activity are significantly enhanced after the application of 600 and 900 μg/mL PCPs (*p* < 0.05). The reactive oxygen species (ROS), the malondialdehyde (MDA) levels, and the glutathione peroxidase (GSH-Px) activity, in comparison with the control group, are significantly decreased in all groups with PCPs (all *p* < 0.05). The enzymatic activity of superoxide dismutase (SOD) in spermatozoa is significantly higher in the treatment with 600 μg/mL of PCPs than in the other groups (*p* < 0.05). As compared with the control group, a significant increase in the catalase (CAT) level is found in the groups with PCPs at 300, 600, 900, and 1200 μg/mL (all *p* < 0.05). In comparison with the control group, the 5-methylcytosine (5-mC) levels are significantly decreased in all groups with PCPs (all *p* < 0.05). As a result of these findings, a certain amount of PCPs (600–900 μg/mL) added to the cryodiluent can significantly improve the quality of Shanghai white pig spermatozoa and can also reduce the methylation of spermatozoa DNA caused by cryopreservation. This treatment strategy may establish a foundation for the cryopreservation of semen from pigs.

## 1. Introduction

Throughout the world, artificial insemination (AI) has been used for many years to successfully conserve animal genetic resources [1]. In recent years, AI with frozen–thawed semen has fulfilled the needs for the reproduction of high-quality pigs; it minimizes the costs for housing or transporting live animals, and it allows for the preservation of high-quality semen specimens for further use. However, the rate of successful conception is still not absolutely satisfactory and is usually lower than that from fresh semen specimens, and this is despite the continuous development and optimization of the cryopreservation technique over the last few decades. In this context, the quality of cryopreserved pig semen will have to improve in order to facilitate the reproduction of high-quality pigs. In semen, the antioxidant system is unable to scavenge for excess reactive oxygen species (ROS) that are important for cell metabolism [2]. In addition, the high-level ROS will make spermatozoa fragile and reduce spermatozoa quality, resulting in a low rate of successful conceptions [3,4]. Moreover, it was reported that the oxidative stress-induced human spermatozoa DNA damage hinders DNA methylation; however, the supplementation of antioxidants might reduce the DNA damage and restore DNA methylation [5]. Therefore, restoring and maintaining the oxidative balance in semen is important during cryopreservation, and this can be achieved through the addition of a moderate amount of antioxidants into the cryodiluent.

In Chinese medicine, *Poria cocos* is called Fuling. It is an edible medicinal fungus that has been used for over two thousand years [6]. Pharmacological studies have shown that polysaccharides are the most abundant substance in *Poria cocos* and have a wide range of biological activities, including anti-inflammatory, immunomodulatory, antiviral, antioxidant, anti-tumor, antidiabetics, and anti-hemorrhagic fever effects [7,8,9]. *Poria cocos* mushroom polysaccharides (PCPs) have been proven to have a suppressive effect on pig spermatozoa DNA methylation when added to a cryodiluent [10]. However, to our knowledge, their effects on pig spermatozoa quality and their antioxidant function have never been reported, and no studies have been published concerning their impact on DNA methylation when applied at varying concentrations during cryopreservation.

In the present study, we aim to explore whether PCPs can be used as a protectant for pig semen during cryopreservation and to determine the optimal amount for the procedure. In the meantime, an analysis of PCP effects on the quality of sperm, antioxidant function, and DNA methylation is also conducted.

## 2. Materials and Methods

### 2.1. Semen Collection

**Figure 1:**
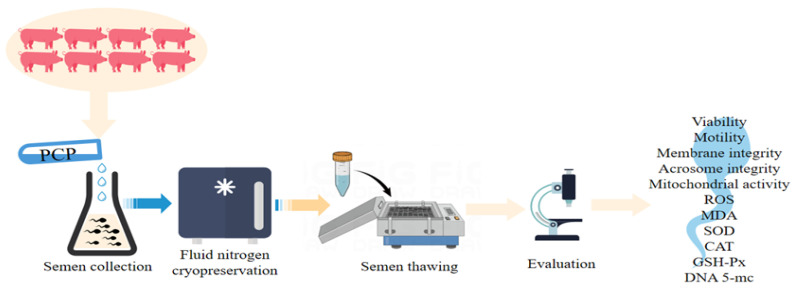
Schematic representation of the experimental schedule.

Eight Shanghai white pigs (boars) aged 1.5–2.5 years and weighing around 240 kg were selected. All the pigs came from the Shanghai Agricultural White Pigs Seed Farm (Shanghai, China). All the pigs were housed in individual pens, fed the same diet, and given drinking water ad libitum every day. The experimental design is shown in Figure 1. A total of 24 ejaculates (three ejaculate samples per boar) were manually collected. The semen samples were simple milky-white in color with no aversive odor or contamination. Following sampling, the samples were immediately wrapped in several layers of gauze, placed in a temperature-controlled incubator at 37 °C, and immediately sent to the laboratory. All the samples fulfilled the given criteria of a volume > 200 mL, a concentration > 2 × 10^8^ spermatozoa/mL, and a viability > 80%, and were used in further experiments [11].

### 2.2. Semen Cryopreservation

The semen samples were instantly equilibrated in a 17 °C refrigerator for 1 h, and then centrifuged at 17 °C and 2060 r/min for 15 min. The supernatant was discarded. The pooled semen samples were equally divided into six groups and frozen in the cryodiluent with different concentrations of PCPs. Solution I was prepared with an 80% mixture (100 mL diluted in double-distilled water): tri-hydroxymethyl aminomethane (2.42 g), citric acid (1.48 g), glucose (1.1 g), penicillin sodium (0.06 g), streptomycin sulfate (0.1 g), and 20% vitelline. Solution I was added to each group, followed by 0, 300, 600, 900, 1200, and 1500 μg/mL of PCPs. The extended samples were placed in a water bath at 17 °C and maintained for about 3 h. Thereafter, the semen was slowly cooled to 4 °C in a refrigerator and kept for a period of 3–4 h for equilibration [12]. At 4 °C, an equal volume of pre-cooled solution II (solution I with an additional 3% of glycerol) was added to each group, followed by 0, 300, 600, 900, 1200, and 1500 μg/mL of PCPs. Eventually, the PCP concentration in each group was 0 (control), 300, 600, 900, 1200, and 1500 μg/mL, respectively. The samples were aspirated into 0.5 mL French straws (IMV, Normandy, France), and were frozen horizontally in liquid nitrogen vapor (4 cm above liquid nitrogen) for 15 min. The frozen samples were stored at −196 °C in liquid nitrogen until their further use.

### 2.3. Semen Assessment after Thawing

The frozen straws (*n* = 30, 5 per group) were placed in a water bath at 60 °C for 12 s. Subsequently, the 100 μL semen samples were mixed with 900 μL of defrosted liquid and then thawed at 37 °C in a water bath for 5 min. The preparation of the defrosted liquid was carried out using 95% Beltsville Thawing Solution (BTS) extender (100 mL diluted in double-distilled water): ethylene diamine tetraacetic acid (EDTA, 0.125 g), trisodium citrate dihydrate (0.6 g), potassium chloride (0.075 g), sodium bicarbonate (NaHCO_3_, 0.125 g), glucose (3.7 g), penicillin sodium (0.06 g), streptomycin sulfate (0.1 g), and 5% of solution I [13]. Then, the quality, antioxidant function, and DNA methylation of the spermatozoa were analyzed.

#### 2.3.1. Viability and Motility of the Spermatozoa

To evaluate the spermatozoa viability, the suspension of spermatozoa was carefully mixed with eosin-y staining (Nanjing KeyGen Biotech Co., Ltd., Nanjing, China) that stained the dead spermatozoa pink while the live spermatozoa remained uncolored. The samples (10 μL) were added to 2 µL eosin-y 0.05% and mixed gently. Then, the samples were observed under an inverted microscope at 400× magnification. It was determined that a minimum of 200 spermatozoa from five different fields were evaluated. A minimum of 200 spermatozoa from four different fields were counted to obtain the percentage of spermatozoa viability.

A computer-assisted semen analyzer (CASA, Version 12.3, Hamilton Thorne, Beverly, MA, USA) was used for the evaluation of the spermatozoa motility. For analysis, aliquots (8 µL) of semen were placed on a pre-warmed chamber slide and the spermatozoa motility characteristics were determined with a 10× objective at 37 °C. For the evaluation of the spermatozoa motility, 10 microscopic fields were analyzed and included at least 300 spermatozoa.

#### 2.3.2. Integrity of the Spermatozoa Acrosome

Fluorescein isothiocyanate-labeled peanut agglutinin (FITC-PNA) was applied to observe the morphology of the spermatozoa acrosomes [14]. Three percent polyvinylpyrrolidone (PVP, 2 mL) was added to the thawed semen samples and centrifuged twice at 800× *g* for 3 min. Upon removing the supernatant, the pellet was resuspended in PBS, and the concentration was modulated to (1–2) × 10^6^ spermatozoa/mL. A smear was made with 30 μL of semen suspension, followed by air-drying and a 10 min fixation with paraformaldehyde. The spermatozoa were incubated with 30 μL of FITC-PNA at 37 °C for 15 min in the dark. Then, the samples were washed with PBS, air-dried, a few brighteners (glycerol:PBS = 9:1) were added, and they were then sealed with a cover slip. The spermatozoa acrosomes were observed under a fluorescence microscope (400× amplification; Nikon Corporation, Tokyo, Japan). Four types of spermatozoa acrosomes could be identified: a, intact spermatozoa acrosomes that displayed a strong fluorescence in the acrosomal region of the spermatozoa and they had regular edges; b, partially damaged spermatozoa acrosomes that displayed a chaotic fluorescence in the anterior acrosomal region and had irregular edges; c, spermatozoa acrosomes without a cap that displayed fluorescence only in the equatorial region; and d, completely damaged spermatozoa acrosomes without any fluorescence. A total of 200 spermatozoa in at least 3 microscope fields were examined, each in duplicate.

#### 2.3.3. Integrity of the Spermatozoa Plasma Membranes

To assess the integrity of the spermatozoa plasma membranes, the hypo-osmotic swelling test (HOST) was carried out [8]. Thawed semen samples were diluted in a fructose–sodium citrate hypotonic solution at a concentration of 1 × 10^6^ spermatozoa/mL. Following pre-incubation at 37 °C for 10 min, 20 μL semen suspension drops were applied to a blood cell counting plate and observed under an inverted microscope at 400× magnification. The percentage of bent-tail spermatozoa over the total spermatozoa number was calculated. A total of 200 spermatozoa in at least 3 microscope fields were examined, each in duplicate.

#### 2.3.4. Mitochondrial Activity

The mitochondrial activity was analyzed using combined propidium iodide (PI) and rhodamine 123 (Rh123) staining. The thawed semen samples were diluted in isothermal BTS at a concentration of (3–6) × 10^6^ spermatozoa/mL. Prior to staining, 1 μL PI and 1 μL Rh123 were incubated in the dark in a centrifuge tube containing 100 μL isothermal HEPES/BSA buffer for 10 min. Subsequently, a 50 μL semen sample was incubated for 30 min at 37 °C in a dark, wet atmosphere. A smear was made with a 10 μL semen suspension, and a few brighteners (glycerol:PBS = 9:1) were added. The sample was mixed, sealed, and observed under an inverted microscope at 400× magnification. As a result, PI-negative and Rh123-positive spermatozoa after UV excitation were identified as live spermatozoa with a high mitochondrial membrane potential. The percentages of viable spermatozoa and spermatozoa with activated mitochondria were calculated. A total of 200 spermatozoa in at least 3 microscope fields were examined, each in duplicate.

#### 2.3.5. Antioxidant Function

The intracellular ROS level in spermatozoa cells was measured using 2’,7’-dichlorodihydrofluorescein diacetate (DCFH-DA; Beyotime, Shanghai, China). First, the semen samples were centrifuged at 800 r/min for 5 min, diluted in 10 mM DCFH-DA to a concentration of 5 × 10^6^ spermatozoa/mL, and incubated at 37 °C for 30 min in the dark. Following washing, a luminometer (BioTek, Winooski, VT, USA) was used for the ROS examination. Each examination consisted of at least 3 technical replicates.

Malondialdehyde (MDA) was examined using thiobarbituric acid reactive substances (TBARS) in a modified microplate. The samples were treated with 5% sodium dodecyl sulfate, boiled with 0.53% thiobarbituric acid (TBA), dissolved in 20% acetic acid (pH 3.5) for 1 h, cooled on ice for 10 min, and centrifuged (1750 r/min for 10 min). The supernatant was used to quantify the MDA (µmol/g) at 540 nm on a microplate spectrophotometer (Shanghai Precision Instruments Co., Ltd., Shanghai, China). Each examination consisted of at least 3 technical replicates.

In this study, the activity of superoxide dismutase (SOD) was assessed using the method of Flohe et al. [15]. The semen samples were incubated in the mixture containing nitroblue tetrazolium (NBT, 0.025 mM), EDTA (0.1 mM), sodium cacodylate buffer (pH 10.0, 50 mM), xanthine (0.1 mM), and xanthine oxidase (0.1 mM) that were, respectively, diluted in 50 mM phosphate buffered sodium (pH 7.0) at 1:5. The SOD activity (U/mL) was determined according to the enzyme concentration with 50% inhibition of the rate of oxidation of NBT, as measured using a spectrophotometer at 560 nm. Each examination consisted of at least 3 technical replicates.

We tested the catalase (CAT) activity according to Coth et al. [16]. In short, a 3 mL semen specimen was incubated for 60 s in a 1.7 mL substrate containing 65 mM hydrogen peroxide in 50 mM PBS (pH 7.0). An additional 0.5 mL of ammonium molybdate (32.4 mM) was added to the reaction solution. The absorbance of hydrogen peroxide was measured at 405 nm using a spectrophotometer. The CAT activity was shown as U/mL. Each examination consisted of at least 3 technical replicates.

The glutathione peroxidase (GSH-Px) activity was determined using Lawrence and Burk’s method [17]. The reaction mixture was composed of NADPH (0.2 mM), sodium azide (1 mM), potassium phosphate buffer (pH 7.0, 50 mM), GSH (1 mM), EDTA (1 mM), H_2_O_2_ (0.25 mM), and oxidized glutathione reductase (1 EU/mL). A reaction mixture of 0.8 mL was added to the enzyme source (0.1 mL) and incubated at 25 °C for 5 min. Then, the reaction was started with the addition of 0.1 mL peroxide solution. The absorbance was measured at 412 nm using a spectrophotometer. Using the micromoles of NADPH oxidized per minute as an indicator, the GSH-Px activity (nmol/L) was measured. Each examination consisted of at least 3 technical replicates.

#### 2.3.6. Spermatozoa DNA Methylation

The thawed semen samples were diluted in PBS diluents (pH 7.2–7.4) at a concentration of 1 × 10^7^ spermatozoa/mL. The spermatozoa suspension was sonicated on ice using a VC130PB ultrasonic processor (30% amplitude; Sonics and Materials Inc., Newtown, CT, USA) for 30 s to release the intracellular components. Thereafter, centrifugation was performed at 2–8 ℃ for about 20 min at 2000–3000 rpm, and the supernatant was collected. A second centrifugation was required in the presence of precipitation for future use. A DNA enzyme-linked immunosorbent assay kit (ELISA) for 5-methylcytosine (5-mC) was used following the manufacturer’s instructions (Jiangsu Meimian Industrial Co., Ltd., Yancheng, China). A microplate reader was used to determine the OD of each well at 450 nm. The spermatozoa DNA methylation was determined by measuring 5-mC according to the standard curve regression equation. Each group underwent the experiment three times.

### 2.4. Statistical Analysis

The data were analyzed using IBM SPSS Statistics 20.0 (SPSS Inc., Chicago, IL, USA). A one-way ANOVA test was used to determine the significant differences between the groups, followed by a post hoc analysis using the LSD multiple comparisons test. The relationship between the spermatozoa quality and DNA methylation was analyzed using a Pearson correlation analysis. All data are expressed as the mean ± S.E.M. A significance value of *p* < 0.05 was set. GraphPad Prism 6 (GraphPad Software Inc., San Diego, CA, USA) was used to generate the histograms.

## 3. Results

### 3.1. Effect of PCPs on the Quality of Cryopreserved Spermatozoa

**Figure 2:**
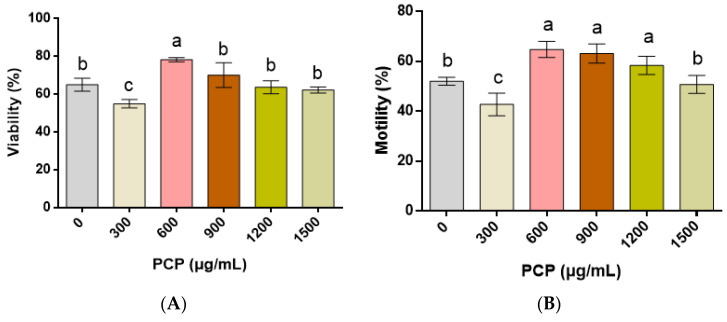
Effect of *Poria cocos* mushroom polysaccharides (PCPs) on the viability (**A**) and motility (**B**) of cryopreserved Shanghai white pig spermatozoa. Values (mean ± S.E.M.) with different letters differ significantly (*p* < 0.05).

In comparison with the control group (0 μg/mL PCP), the application of 600 μg/mL PCPs significantly enhances the post-thaw spermatozoa viability (*p* < 0.05); however, no significant differences in the spermatozoa viability are demonstrated when PCPs re applied at 900, 1200, and 1500 μg/mL (all *p* > 0.05; Figure 2A). In addition, the application of 300 μg/mL PCPs significantly decreases the spermatozoa motility as compared with those in the other groups (*p* < 0.05; Figure 2B). In terms of the spermatozoa motility, it is significantly enhanced after treatment with 600, 900, and 1200 μg/mL of PCPs, whereas it is weakened when 300 μg/mL of PCPs is applied (all *p* < 0.05).

**Figure 3:**
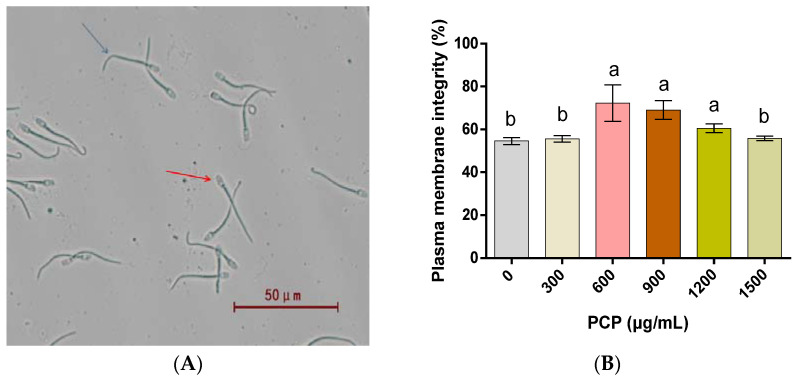
Effect of *Poria cocos* mushroom polysaccharides (PCPs) on the integrity of the plasma membranes of cryopreserved Shanghai white pig spermatozoa. (**A**) Arrow marks indicate the positive (intact plasma membranes, blue arrow) hypo-osmotic swelling test (HOST) and the negative (damaged plasma membranes, red arrow) HOST sperm, (**B**) the percentage of viable spermatozoa with intact cell membranes. Values (mean ± S.E.M.) with different letters differ significantly (*p* < 0.05).

**Figure 4:**
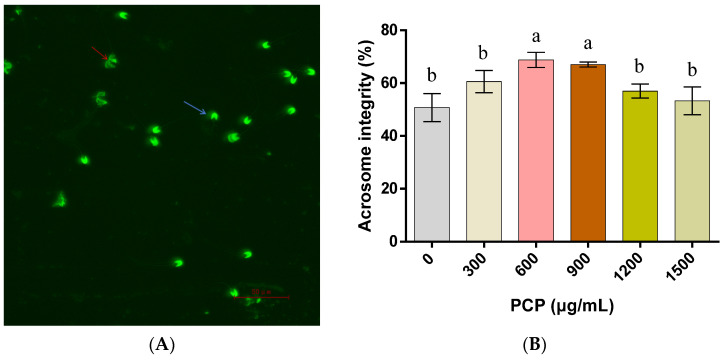
Effect of the *Poria cocos* mushroom polysaccharides (PCPs) on the integrity of the acrosomes of cryopreserved Shanghai white pig spermatozoa. (**A**) Spermatozoa with intact (blue arrow) or non-intact (red arrow) acrosomes stained with fluorescein isothiocyanate-labeled peanut agglutinin (FITC-PNA), (**B**) the percentage of spermatozoa with intact acrosomes. Values (mean ± S.E.M.) with different letters differ significantly (*p* < 0.05).

**Figure 5:**
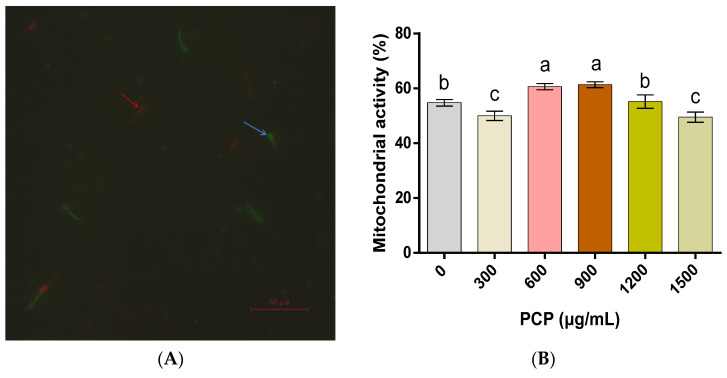
Effect of *Poria cocos* mushroom polysaccharides (PCPs) on the mitochondrial activity of cryopreserved Shanghai white pig spermatozoa. (**A**) Spermatozoa mitochondrial activity assessed through staining with propidium iodide (PI) and rhodamine 123 (Rh123). Blue arrow indicates intact mitochondrial membrane potential; red arrow indicates compromised mitochondrial membrane potential, (**B**) the percentage of mitochondrial activity of the spermatozoa. Values (mean ± S.E.M.) with different letters differ significantly (*p* < 0.05).

In regard to the integrity of the plasma membranes of spermatozoa (Figure 3), PCPs at 600, 900, and 1200 μg/mL contribute to more intact spermatozoa plasma membranes (*p* < 0.05), while PCPs at 300 and 1500 μg/mL show no significant effect (*p* > 0.05). With respect to the integrity of the spermatozoa acrosomes, almost no significant differences are found before and after the PCP application, in addition to a significant increase after treatment with 600 and 900 μg/mL of PCPs (*p* < 0.05, Figure 4). As for the mitochondrial activity of the spermatozoa, it is significantly increased with 600 and 900 μg/mL of PCPs (*p* < 0.05), marginally varies after the application of 1200 μg/mL PCPs (*p* > 0.05), and becomes remarkably lower when 300 and 1500 μg/mL of PCPs are applied (*p* < 0.05, Figure 5).

### 3.2. Effect of PCPs on the Antioxidant Function of Cryopreserved Spermatozoa

**Figure 6:**
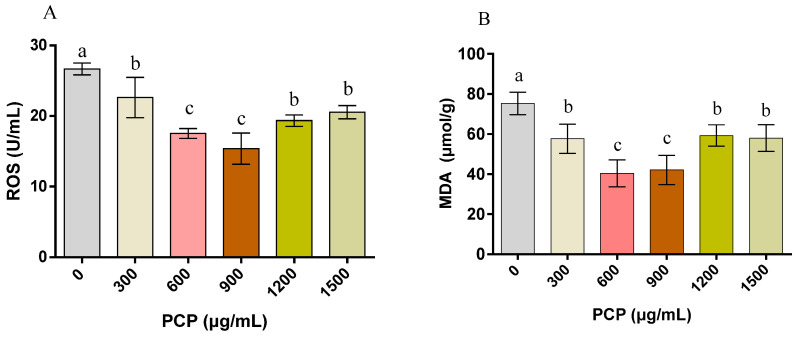

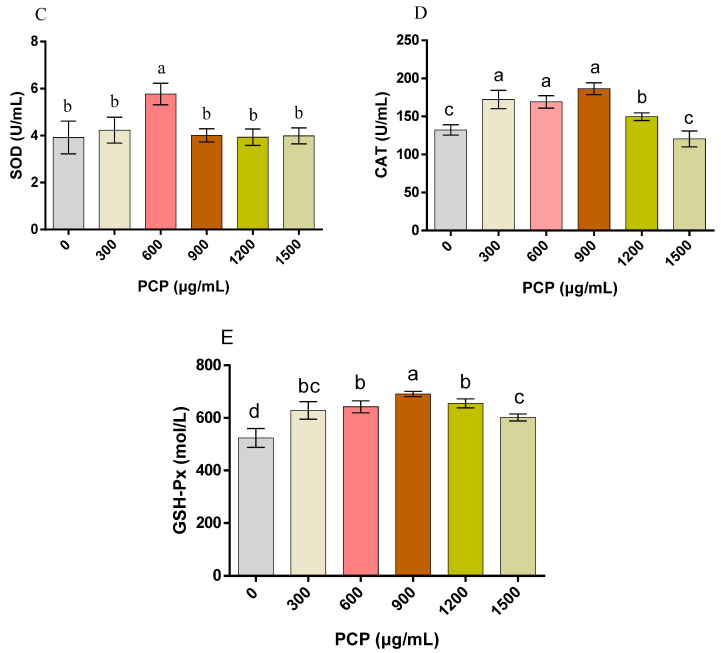
Effect of *Poria cocos* mushroom polysaccharides (PCPs) on the antioxidant function of cryopreserved Shanghai white pig spermatozoa. (**A**) Reactive oxygen species (ROS), (**B**) malondialdehyde (MDA), (**C**) superoxide dismutase (SOD), (**D**) catalase (CAT), and (**E**) glutathione peroxidase (GSH-Px) of the spermatozoa. Values (mean ± S.E.M.) with different letters differ significantly (*p* < 0.05).

The effect of PCPs on the frozen–thawed spermatozoa antioxidant function is shown in Figure 6. Significant reductions in ROS and MDA levels are found in all groups with PCPs (*p* < 0.05), and the reduction is much more obvious in groups with PCPs at 600 and 900 μg/mL. The enzymatic activity of SOD in spermatozoa is significantly higher in the treatment with 600 μg/mL of PCPs (*p* < 0.05) than in the other groups. As compared with the control group, a significant increase in the CAT level is found in groups with PCPs at 300, 600, 900, and 1200 μg/mL (all *p* < 0.05), but no obvious change is found after the application of 1500 μg/mL PCPs (*p* > 0.05). It is of note that the GSH-Px activity in spermatozoa increases significantly in all groups with PCPs (all *p* < 0.05), especially in groups with PCPs at 900 μg/mL.

### 3.3. Effect of PCPs on the Spermatozoa DNA Methylation

**Figure 7:**
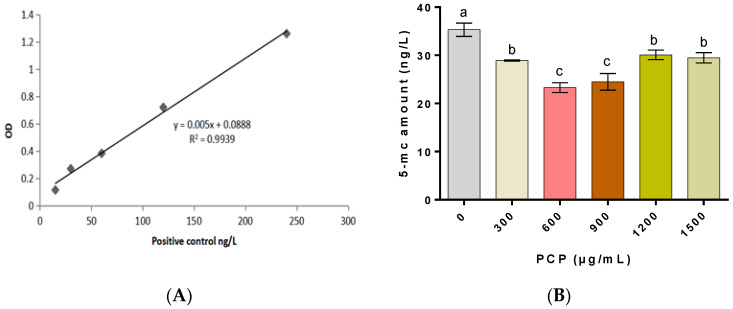
(**A**) Standard curve for the measurement of 5-mC, (**B**) 5-mC amount representing spermatozoa DNA methylation in each group. Different lowercase letters indicate significant differences (*p* < 0.05).

With various levels of 5-mC (5, 15, 30, 60, 120, 140 ng/L), a standard curve was constructed. As shown in Figure 7A, a linear analysis was performed with five points, and a fitting curve of y = 0.005x + 0.0888 was generated (R_2_ = 0.9939). The fitting curve performs well with reliable results, based on which 5-mC can be measured. Figure 7B shows that the 5-mC levels are significantly lower in PCP groups than in the control groups (all *p* < 0.05), while they re much lower in the groups with PCPs at 600 and 900 μg/mL than in the groups with PCPs at 300, 1200, and 1500 μg/mL (all *p* < 0.05).

The relationship between the spermatozoa quality and DNA 5-mC was analyzed after the PCP application. As revealed in Table 1, remarkable negative associations are found between spermatozoa DNA 5-mC and spermatozoa viability (R = −0.567), motility (R = −0.553), and mitochondrial activity (R = −0.496) (all *p* < 0.05). Moreover, the spermatozoa DNA 5-mC also shows strong negative associations with the integrity of spermatozoa plasma membranes (R = −0.789) and spermatozoa acrosomes (R = −0.769) (both *p* < 0.05).

## 4. Discussion

### 4.1. Effect of PCPs on the Quality of Cryopreserved Shanghai White Pig Spermatozoa

As confirmed by existing studies, PCPs derived from the Chinese herbal medicine Fuling have a strong antioxidant capacity [18]. Currently, multiple Chinese herbal medicines, such as *Salvia miltiorrhiza* polysaccharides, Rhodiola polysaccharides, and Laminaria japonica polysaccharides, have been confirmed to have a protective effect on spermatozoa during cryopreservation [19,20]. However, there are no previous reports on the effect of different concentrations of PCPs on pig spermatozoa during cryopreservation.

The aim of this study is to evaluate the spermatozoa quality of Shanghai white pigs after the cryopreservation of semen in diluents with different concentrations of PCPs. In the meantime, this study intends to gain more information about the antioxidant capabilities of PCPs. The results reveal that PCPs can significantly improve almost all spermatozoa quality parameters after freezing and thawing.

The motility and viability of spermatozoa are important indicators for assessing the quality of spermatozoa, and the spermatozoa motility is tightly linked to fertilization results [21]. Our study finds that PCPs at 600 μg/mL perform the best in preserving the spermatozoa motility and viability. A previous study reveals that the addition of an appropriate amount of Rhodiola polysaccharides in a cryoprotectant has positive effects on the motility of pig spermatozoa, which is consistent with the findings in our study [22]. This might be due to the protective effect of polysaccharides, which are non-penetrating cryoprotectants, on spermatozoa by preventing dehydration during cryopreservation, as they prevent the formation of ice crystals within the spermatozoa and then improve their motility. This study also notes that PCPs at 1500 μg/mL result in significant reductions in the spermatozoa motility and viability, in comparison with PCPs at the lower concentration of 600 μg/mL. There may be two reasons: a, the PCP concentration is too high and produces osmotic stress, which destroys the normal structure of the spermatozoa and then reduces the spermatozoa viability after thawing; or b, the PCP concentration is too high and also induces cytotoxicity, which accelerates the apoptosis of spermatids and shortens the survival time, eventually leading to a decline in spermatozoa quality.

Spermatozoa acrosomes contain a number of hydrolytic enzymes that serve as the structural basis for spermatozoa to undergo an acrosome reaction. The spermatozoa plasma membranes are important for maintaining the balance between the intracellular and extracellular compartments; moreover, they also participate in the acrosomes’ reaction during spermatozoa capacitation [23]. The results of this study reveal that PCPs at a range of 600–900 μg/mL preserve the spermatozoa acrosomes and plasma membranes well after freezing and thawing, suggesting a protective effect of PCPs. Similarly, the study of Zhao et al. reports that the supplementation of Astragalus polysaccharides for semen cryopreservation significantly improves the integrity of the acrosomes and plasma membranes of pig semen [10]. Yang et al. also confirm that the addition of Rhodiola rosea polysaccharides in a cryodiluent at a certain concentration distinctly increases the integrities of spermatozoa acrosomes and plasma membranes [20]. As other polysaccharides, PCPs also help preserve the integrity of spermatozoa acrosomes via their functions in antioxidation and in stabilizing phospholipid molecules, thereby improving the quality of frozen spermatozoa. Additionally, PCPs have protective effects on the cell membrane, which this may be caused by two characteristics: the first is the self-oxidation resistance property of PCPs that reduces the ROS content in spermatids, and then preserves the integrity of the cell membrane; the second is the nature of PCPs, which are hydroxyl groups that exert a certain role in stabilizing phospholipid molecules, the main components of a cell membrane.

An intact mitochondrial membrane structure is the basis for the normal oxidative phosphorylation in spermatozoa. It has been reported that the mitochondrial activity of boar spermatozoa decreases after freezing and thawing, resulting in a decreased rate of successful fertilization [24]. This study finds that PCPs at 600–900 μg/mL significantly enhance the mitochondrial activity of Shanghai white pig spermatozoa. There are no references on the effect of different concentrations of PCPs on pig spermatozoa during cryopreservation. However, some scholars have found that the addition of other types of polysaccharides in pig semen improves the quality of spermatozoa. For instance, Yang et al. prove that the addition of Rhodiola rosea polysaccharides to a cryoprotectant significantly increases the mitochondrial activity of frozen–thawed boar spermatozoa [20]. Consistently, our study notes that the addition of PCPs into the cryodiluent improves the quality of frozen–thawed spermatozoa.

### 4.2. Effect of PCPs on the Antioxidant Function of Cryopreserved Shanghai White Pig Spermatozoa

ROS are byproducts of the normal oxidative metabolism in the body, and they are crucial for the cellular metabolism and for maintaining homeostasis. The pattern of MDA directly reflects the level of lipid peroxidation and indirectly reflects the extent of cell damage [24]. An increase in the SOD activity implies a stronger ability to resist damage from free radicals [25]. CAT is an enzyme widely present in various organisms, and a higher content of CAT in cells suggests a stronger antioxidant capacity of the cells. As a catalyst for H_2_O_2_ in semen, GSH-Px protects the integrity of the structure and function of the cell membrane. This research performs studies on the five molecules: ROS, MDA, SOD, CAT, and GSH-Px, to analyze the oxidative damage to pig spermatozoa during cryopreservation. Our results show that PCPs ranging from 600 to 900 μg/mL significantly reduce the concentration of ROS and MDA in cryopreserved spermatozoa. This finding suggests that the supplementation of PCPs into the protectant may reduce the autoxidation of the lipid membrane and subsequently the concentration of ROS and MDA. Zhao et al. report that PCPs can significantly reduce the ROS and MDA produced by the oxidized low-density lipoprotein (ox-LDL)-induced oxidative stress [18]. Another study by Chuang-Ye Li et al. proves that HK-2 cells treated with PCPs experience less oxidative damage with increased cell viability and SOD activity, while the levels of ROS and MDA decrease [25]. Consistently, this study proves reductions in ROS and MDA after PCP application. Ren et al. find that supplementation of *Isatis indigotica* polysaccharides at a certain concentration in a cryodiluent effectively eliminates the ROS and MDA in pig spermatozoa produced during freezing and thawing [26]. Additionally, Yan et al. also confirm that *Isatis lycium barbarum* polysaccharides added into a diluent for human semen cryopreservation reduce the production of ROS and MDA during freezing and thawing [27]. These findings agree with the results of this study. The application of the proper amounts of PCPs or other types of polysaccharides has a certain protective effect on cryopreserved spermatozoa, which may be due to the reduction in lipid oxidation after the polysaccharide binding with the spermatozoa plasma membrane.

In this study, it is found that PCPs at 600 μg/mL contributes the most to the SOD enzymatic activity in cryopreserved spermatozoa, in comparison with other concentrations of PCPs. This may be due to the function of PCPs in ROS scavenging. In detail, several studies have proven that PCPs have the ability to scavenge ROS, NO, and OH, providing direct antioxidant activity to relieve oxidative stress.

In the study by Shen et al., a significant increase in the SOD activity is reported in cryopreserved boar spermatozoa after the addition of *Salvia miltiorrhiza* polysaccharides at 0.2–0.6 mg/mL [19]. Moreover, Hu et al. find that Laminaria japonica polysaccharides also enhance the SOD enzymatic activity in the cryopreserved spermatozoa of pigs [28].

This study also notes that PCPs ranging from 300 to 900 μg/mL significantly increase the CAT activity in cryopreserved spermatozoa, while PCPs at higher concentrations (900–1200 μg/mL) enhance the GSH-Px activity in spermatozoa during cryopreservation. The similarity between PCPs and some antioxidants may be the reason for this result. PCPs protect spermatozoa by scavenging superoxide anion groups and inhibiting lipid peroxidation, which is similar to the function of some other polysaccharides [29]. For example, Shen et al. find that *Salvia miltiorrhiza* polysaccharides supplemented in a cryodiluent remarkably increase the activity of CAT in pig spermatozoa [19]. Zhang et al. note that Rhodiola polysaccharides not only efficiently improve the quality of frozen–thawed pig spermatozoa, but also increase the GSH-Px activity [22]. Hu et al. also report that Laminaria japonica polysaccharides, at a certain concentration, significantly enhance CAT and GSH-Px activities in the cryopreserved spermatozoa of pigs [28]. All of these findings together indicate that PCPs protect frozen–thawed spermatozoa from ROS damage, thereby enhancing the antioxidant defense of the spermatozoa during cryopreservation.

### 4.3. Effect of PCPs on the DNA Methylation of Cryopreserved Shanghai White Pig Spermatozoa

As a potent epigenetic mechanism, DNA methylation is an important regulator of the expression levels of coding genes [30]. During spermatogenesis, DNA methylation is indispensable for maintaining the silencing of transposons before meiosis [31]. It has been reported that the aberrant DNA methylation pattern in spermatozoa has some correlation to infertility [32]. Aberrant DNA methylation can cause reduced spermatozoa motility and failure of embryonic development, and more critically, it can induce an epigenetic disease in the offspring. Therefore, DNA methylation is critical for the normal morphology and function of spermatozoa [33]. In the study by Aurich C et al., 5-mC was quantitatively analyzed via ELISA, and the result suggests an increased level of DNA methylation in the spermatozoa of horses after freezing [34]. Consistently, Yeste et al. also confirm that cryopreservation enhances the DNA methylation of spermatozoa in boars [35]. This finding shows that the appropriate concentration of PCPs in semen cryopreservation significantly reduces the effect of cryopreservation on the DNA methylation in pig spermatozoa. Similarly, Rhodiola polysaccharides are also reported to decrease the effect of cryopreservation on the DNA methylation in Tibetan wild boar spermatozoa [36]. However, it is still unclear how PCPs restore the DNA methylation level after cryopreservation. There is an opposite conclusion that cryopreservation has no effect on the DNA methylation in human spermatozoa. This may be attributed to multiple factors, such as the different species and experimental conditions, and further research is warranted. Moreover, this study also analyzes the relationship between various quality parameters and the DNA methylation in pig spermatozoa, and significant negative correlations are reported.

## 5. Conclusions

Overall, this study confirms the suitable PCP quantity (600–900 μg/mL) to add to a cryodiluent for spermatozoa cryopreservation, not only to improve the post-thawed spermatozoa quality and prevent membrane damage, but also to improve the redox balance activity and the DNA methylation levels. Further studies are required to confirm these findings and to investigate the effect of PCP inclusion on the fertility rate of pigs.

## Figures and Tables

**Table 1:** Relationship between the spermatozoa DNA methylation and quality.

Target	Viability	Motility	Plasma Membrane Integrity	Acrosome Integrity	Mitochondrial Activity
Spermatozoa DNA methylation	−0.567 *	−0.553 *	−0.789 **	−0.769 **	−0.496 *

Note: * indicates significant correlation (*p* < 0.05), ** indicates very significant correlation (*p* < 0.01).

## Data Availability

Not applicable.
